# The New Local Anæsthetic

**Published:** 1885-01

**Authors:** G. L. Curtiss

**Affiliations:** Syracuse, N. Y.


### THE NEW LOCAL ANÆSTHETIC.

BY G. L. CURTISS, D. D. S., SYRACUSE, N. Y.

In the last number of the INDEPENDENT PRACTITIONER reference is made to the experiments upon sensitive dentine of Dr. Julian W. Russell, with the new local anæsthetic, Hydrochlorate or Muriate of Cocaine. (Muriatic is but another name for Hydrochloric acid.)

I, too, have been using the drug since its first appearance in this country, and with very satisfactory results, insensibility of tissue usually taking place in from three to five minutes, and lasting from ten to fifteen. I have observed that the best results are seen in soft teeth, of a loose structure. My experiments have been confined to the use of the drug as an obtunder of sensitive dentine, and I find that excavation can, under its influence, be accomplished with little or no pain to the patient. I have also used it upon the gums before lancing or scarifying. I had prepared a more elaborate report of my experience, but I presume the points will be covered by the paper of Dr. Russell.

